# The Many Roles of Dermoscopy in Melanoma Detection

**DOI:** 10.3390/life13020477

**Published:** 2023-02-09

**Authors:** Cristina-Raluca (Jitian) Mihulecea, Gabriela Mariana Iancu, Mihaela Leventer, Maria Rotaru

**Affiliations:** 1 Doctoral Studies, Victor Babeș University of Medicine and Pharmacy of Timișoara, 300041 Timișoara, Romania; 2 Dermatology Clinic, Emergency Clinical County Hospital of Sibiu, 550245 Sibiu, Romania; 3 Dermatology Department, Faculty of Medicine, Lucian Blaga University of Sibiu, 550169 Sibiu, Romania; 4 Dr Leventer Centre—Dermatology Clinic, 011216 Bucharest, Romania

**Keywords:** nevi, melanoma, dermoscopy, melanomagenesis, tumoral thickness

## Abstract

Dermoscopy is a non-invasive method of examination that aids the clinician in many ways, especially in early skin cancer detection. Melanoma is one of the most aggressive forms of skin cancer that can affect individuals of any age, having an increasing incidence worldwide. The gold standard for melanoma diagnosis is histopathological examination, but dermoscopy is also very important for its detection. To highlight the many roles of dermoscopy, we analyzed 200 melanocytic lesions. The main objective of this study was to detect through dermoscopy hints of melanomagenesis in the studied lot. The most suspicious were 10 lesions which proved to be melanomas confirmed through histopathology. The second objective of this study was to establish if dermoscopy can aid in estimating the Breslow index (tumoral thickness) of the melanomas and to compare the results to the histopathological examination. We found that the tumoral thickness may be estimated through dermoscopy, but the histopathological examination is superior. To conclude, the aim of this study was to showcase the versatility and many roles of dermoscopy, besides being one of the most important tools for early melanoma diagnosis.

## 1. Introduction

Dermoscopy is a non-invasive method of examination that aids the clinician in numerous ways, especially in the establishment of an early skin cancer diagnosis, significantly increasing the accuracy of melanoma detection. Besides the classification of melanocytic lesions, dermoscopy may also be used to predict the thickness of tumors based on specific dermoscopic criteria and colors, which could aid in choosing the right treatment option (dermoscopy is highly effective in skin cancer surgery, as it can help in preoperative marking and the selection of surgical margins) [1].

The main melanocytic lesions are nevi and melanoma. Melanocytic nevi can be classified as follows: acquired nevi (dysplastic—junctional/lentiginous, or compound, Spitz, and Reed), congenital nevi (superficial, superficial and deep, dermal—Miescher (located on the face), and Unna (located on the body)) [1]. The features of Spitz nevi often make them difficult to differentiate from melanomas [2]. They can affect individuals of all ages, but usually appear during childhood (mostly affecting children under 10 years old) or develop in young adults [2]. The management of these lesions is difficult due to their similarity to melanoma.

Melanoma is considered one of the most aggressive forms of skin cancer worldwide, affecting individuals of any age. It has a high potential to metastasize and is responsible for over 80% of deaths caused by skin cancers [3]. Cutaneous melanoma may be classified into the following subtypes: superficial spreading (considered to develop mostly on preexisting nevi), nodular, lentigo maligna, acral lentiginous (higher incidence in dark-skinned patients), amelanotic, and desmoplastic (rare subtype found in older individuals—formed by scant spindle cells with minimal cellular atypia) [1,4]. Tumoral thickness (Breslow index) is one of melanoma’s most important prognostic factors [3]. The patients’ prognosis is strongly related to the early diagnosis of melanoma, and their survival is inversely proportional to the tumoral thickness [3]. The Breslow index is measured through the histopathological examination, but an estimation of the tumoral thickness can also be made through dermoscopy (colors, dermoscopic structures). It may be difficult to differentiate thin melanomas from atypical nevi, as they can have similar clinical, dermoscopic, and even histopathologic characteristics. Certain studies attest to a linear progression, from nevi, atypical nevi, to melanoma, that may occur under mutational factors that are not yet fully understood [5]. This linear progression, although rare and controversial, is stated to apply to melanomas developing on preexisting nevi (20–30% are nevus-associated melanomas (NAM)), as the majority of melanomas develop de novo (approximately 70% are de novo melanomas (DNM)) [4,6,7,8]. Dermoscopy can help discover hints of melanomagenesis and may also aid in differentiating between DNM and NAM [6,7,8]. Besides the assessment of skin tumors (for patient monitoring, early skin cancer diagnosis, estimation of tumoral thickness), dermoscopy can also be used to examine perilesional skin (for example, for assessing solar damage), inflammatory conditions, and dermatoses (psoriasis, dermatitis—which can help differentiate these conditions from actinic keratoses or an in situ squamous cell carcinoma), hair and scalp (trichoscopy), skin infestations and infections (entomodermatoscopy), and nails [1].

The main objective of this study was to detect through dermoscopy hints of melanomagenesis in the studied melanocytic lesions and to showcase the versatility and many roles of dermoscopy, besides being one of the most important tools for early melanoma diagnosis. The second objective of this study was to establish if dermoscopy can aid in estimating the Breslow index (tumoral thickness) of the melanomas and to compare the results to the histopathological examination. We found that the tumoral thickness may be estimated through dermoscopy, but the histopathological examination remains superior. To conclude, dermatoscopy is an essential tool for dermatologists, helping them read the “messages” left by skin cancers and many other dermatological conditions.

## 2. Materials and Methods

We examined 200 melanocytic lesions (dermoscopic images) of dermatologically monitored patients between 2017 and 2022 at the Emergency Clinical County Hospital of Sibiu and a private dermatology office from Sibiu, Romania. We assessed the dermoscopic images to detect any hints of melanoma. The most suspicious lesions were either excised or proposed for excision. Out of all the studied lesions, 10 proved to be melanomas, which were histopathologically confirmed. We also tried to determine the tumoral thickness of the melanomas to establish if dermoscopy can be used in this regard, and we compared our findings with the histopathological results. The main objective of this study was to detect through dermoscopy hints of melanomagenesis in the studied melanocytic lesions and to showcase the versatility and many roles of dermoscopy, besides being one of the most important tools for early melanoma diagnosis. The study has the approval of the Ethics Committee of Sibiu’s County Clinical Hospital (Sibiu, Romania).

Statistical analysis: This was a retrospective and descriptive study. Data were collected and tabulated on Microsoft Excel spreadsheets for statistical analysis [calculation of the prevalence of the variables (%)]. The variables were expressed in numbers and percentages to simplify the statistical process.

## 3. Results

The results of the present study will be separated into two categories: melanoma (main dermoscopic criteria, dermoscopic prediction of melanoma thickness) and nevi dermoscopic classification (pattern analysis, atypical nevi—dermoscopic key findings of melanomagenesis). The Seven-Point Checklist dermoscopic algorithm [1] (major criteria—atypical pigment network, blue-white veil, atypical vascular pattern—2 points each; minor criteria—irregular streaks (or pseudopods), irregular dots/globules, eccentric hyperpigmentation region (irregular pigmentation), regression structures—1 point each; ≥3 points = melanoma, <3 = nevi) and pattern analysis (reticular, globular, homogenous, starburst patterns, parallel furrow, parallel ridge, lattice-like and fibrillar) were used to analyze the selected lesions for this study [1,9]. While assessing the melanocytic nevi, we highlighted the main dermoscopic criteria that are usually found in melanomas to showcase the similarity between nevi and melanomas. As for the confirmed melanoma lesions, the tumoral thickness was assessed through dermoscopic colors and specific criteria and compared to the histopathological results.

### 3.1. Melanoma—Main Dermoscopic Criteria

Cutaneous melanoma may be classified into the following subtypes: superficial spreading, nodular, lentigo maligna, acral lentiginous, amelanotic, and desmoplastic (rare subtype formed by scant spindle cells with minimal cellular atypia) [4]. The dermatoscopic diagnosis of melanoma is based on the recognition of its chaotic appearance and morphological asymmetry and/or one or more of the following characteristics: atypical network, irregular blotch, irregular dots/globules, irregular streaks/pseudopods, regression structures, white shiny streaks, blue-white veil, atypical vascular pattern, irregular hyperpigmented areas, prominent skin markings, and polygons/angulated lines [10]. Other dermoscopic criteria that may be found in melanomas are the rainbow pattern (a sign of invasive melanoma) [11], rosettes (unknown mechanism) [12], and crusts/erosions (a sign of advanced lesions) [8].

We will list the main dermoscopic criteria that were found while examining the selected melanoma lesions from this study, and they will also be assessed with the Seven-Point Checklist dermoscopic algorithm.

- **Superficial Spreading Melanoma (SSM)**

Five out of the ten melanoma lesions selected for this study were superficial spreading melanomas. All the tumors had morphological asymmetry and the following dermoscopic criteria: regression structures—80%, irregular hyperpigmented areas, atypical network, blue-white veil—60%, pseudopods, polygons/angulated lines, white shiny streaks, irregular dots/globules, crusts/erosions, rosettes—40%, prominent skin markings, rainbow pattern—20%. As for the Seven-Point Checklist dermoscopic algorithm, one of them had 2 points, while four out of five tumors had over 3 points (confirmed melanomas), with one tumor having the highest score of 8 points.

- **Nodular Melanoma (NM)**

Only one tumor was a nodular melanoma. Conventional melanoma dermoscopic findings are typically not found in nodular melanomas as these criteria are specific for this type of tumor: blue and black color (blue-black rule), atypical vascular pattern (linear irregular vessels/more than two types of vessels), milky-red color [10]. The studied tumor had a symmetrical shape and the following dermoscopic criteria: blue-white veil, white shiny streaks, and crusts/erosions. While performing the Seven-Point Checklist dermoscopic algorithm, we obtained a score of 2 points for this lesion.

- **Lentigo Maligna Melanoma (LMM)**

**Figure 1:**
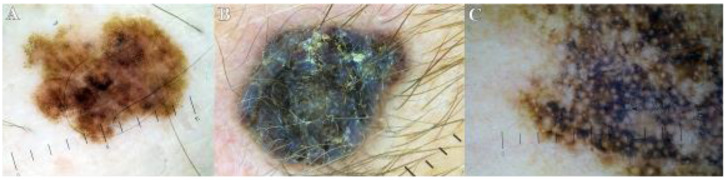
Main melanoma subtypes. (**A**). Superficial spreading melanoma (SSM). (**B**) Nodular melanoma (NM). (**C**). Lentigo maligna melanoma (LMM).

Four out of ten melanomas corresponded to the lentigo maligna subtype. All tumors had an asymmetric morphology, and out of the conventional melanoma dermoscopic criteria, the following were more specific for LMM: irregular hyperpigmented areas, irregular dots/globules, white shiny streaks, regression structures—100%, irregular blotch, polygons/angulated lines, blue-white veil—75%, rosettes—50%, atypical vascular pattern—25%. All tumors had 3 points or more after being assessed with the Seven-Point Checklist dermoscopic algorithm, with 5 points being the highest score, see Table 1—Melanoma—Dermoscopic findings, and Figure 1—Main melanoma subtypes.

- **Dermoscopic prediction of melanoma thickness**

Dermoscopy helps increase the diagnostic accuracy of melanoma and may be of help in estimating the tumoral thickness [13]. Each dermoscopic color has a histopathological correspondent that can help estimate the depth of the pigments and the tumoral thickness: black—melanin found in the stratum corneum, dark/light brown—melanin found in the epidermis or the dermal/epidermal junction, grey—melanin found in the dermis (mostly superficial dermis), blue—melanin found in the deep dermis, yellow/orange—serum/keratin in the epidermis, white—fibrosis, collagen located in the dermis, red—blood found in the vessels in the superficial dermis, purple—low oxygen levels in the blood vessels (the color of blood varies from red to purple/blue, depending on the degree of oxygenation)—located in the deep dermis [1,10]. One of the most important prognostic factors for melanoma is the Breslow index (IB) [3]. To estimate it, we used the correlation of the Clark staging to the IB [14] based on the dermoscopic criteria listed in Table 1. As a correlation of Clark–Breslow index staging (which may vary according to the localization of the tumor and the thickness of the skin of different anatomical sites), we will refer to it as Clark Level 1 (epidermis)—IB ≤ 1.00 mm, Clark Level 2 (papillary dermis)—IB 1.00–2.00 mm, Clark Level 3 (papillary dermis-reticular junction)—IB 2.00–4.0 mm, Clark Level IV (reticular dermis)—IB > 4.0 mm, Clark Level V (subcutaneous invasion). We also assessed the dermatoscopic colors (black, brown, blue, grey, yellow/orange, red, violet) of the studied melanomas, and the following results were obtained:

- **Superficial spreading melanoma (SSM)**

The most frequently encountered dermoscopic colors in this subtype were: black and brown—100%, white—80%, grey and blue—60%, and yellow/orange—40%. To estimate the Breslow index, we assessed the melanomas that had five colors (black, brown, grey, blue, and white) and the highest score (8 points) at the Seven-Point Checklist algorithm. The black/brown colors encountered in this tumor are associated with melanin located in the epidermis, whereas the blue color is associated with melanin found in the deep dermis [1,10]. As for the dermoscopic structures, this tumor had: atypical pigmented network/eccentric hyperpigmentation—mostly correlated with elongated rete ridges with melanin found at the dermal-epidermal junction (DEJ) [15]; blue-white veil—typical to an elevated part of the lesion and its histologic correspondent are extremely pigmented atypical melanocytes/melanophages located in the dermis; pseudopods—associated with heavily pigmented nests of melanocytes mostly located in the DEJ or superficial dermis; irregular dots/globules—melanin located in the epidermis [15]; regression structures—associated with fibrosis located in the dermis [15]. If we were to consider the dermoscopic colors, with blue meaning melanin located in the dermis, the tumor would have a Clark Level IV—IB > 4.0 mm [1,10,14,15]. As for the dermoscopic structures, the tumors should be located somewhere between the epidermis and superficial dermis, meaning a Clark Level of I-II—IB ≤ 1.00, 1.1–2.00 mm [12,13]. The histopathological results of this tumor showed the following: Clark Level II, IB = 0.5 mm, with a pT1a stage.

- **Nodular melanoma (NM)**

We observed the following colors in the assessed nodular melanoma: black, brown, blue, yellow/orange, and white. The correspondents of the tumor’s colors were: black/brown—associated with melanin located in the epidermis, yellow/orange—serum/keratin in the epidermis, blue—melanin found in the deep dermis, white—fibrosis, collagen located in the dermis. The blue-white veil dermoscopic criterion is associated with atypical melanocytes/melanophages in the dermis [15]. Based on these dermoscopic findings, the tumoral thickness should correspond to a Clark Level I-II (epidermis, papillary dermis) = IB 1.00–2.00 mm. The histopathological results showed: Clark Level II, IB of 1.2 mm, and a pT2a stage.

- **Lentigo maligna melanoma (LMM)**

**Figure 2:**
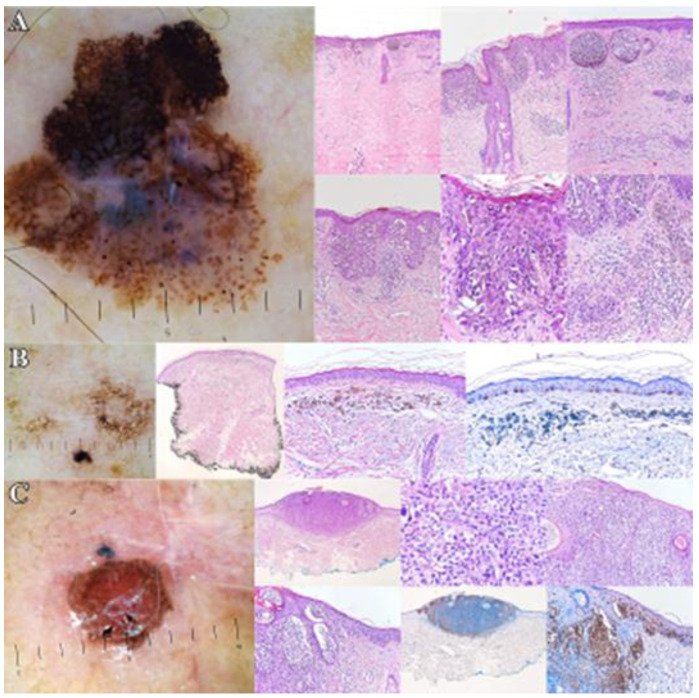
Dermoscopic and histopathologic aspects of melanoma. (**A**). Superficial spreading melanoma (SSM)—Main dermoscopic criteria—asymmetrical melanocytic lesion, with atypical network, eccentric hyperpigmented areas, irregular dots/globules, irregular streaks/pseudopods, central blue-white veil, regression structures. Histopathologic description—SSM without ulceration, BI (Breslow index) = 0.5 mm (pT1a), Clark level II, developed on a preexisting nevus—atypical melanocytes located in the dermal-epidermal junction, with pagetoid ascension. (**B**). Lentigo maligna melanoma (initial lesion)—Main dermoscopic criteria—asymmetrical melanocytic lesions with irregular dots/globules, eccentric hyperpigmented area, and regression structures. Histopathologic description: complete regression of the lentiginous component of a lentigo maligna melanoma—flattened epidermis, severe dermal solar elastosis, melanophages deposited in a band-like in the papillary dermis. Immunohistochemistry—Melan A and SOX 10—melanocytes with quasi-normal characteristics and disposition in the basal layer of the epidermis. (**C**). Lentigo maligna melanoma (vertical growth phase, recurrence after 8 months on the post-operative scar)—Main dermoscopic criteria—atypical vascular pattern, eccentric hyperpigmented area, regression structures. Histopathologic description—LMM (vertical growth phase) without ulceration, BI = 2.2 mm (pT3a), Clark level IV—atypical melanocytes arranged lentiginously and in nests at the dermo-epidermal junction, with pagetoid invasion in the epidermis. Immunohistochemistry—Melan A and Tyrosinase—positive. Histopathologic images—provided through the courtesy of Dr. Tiberiu Tebeică, Dr. Leventer Centre—Bucharest, Romania.

The main colors found in these tumors were black, brown, grey, white—100%, and blue—66.66%. We assessed the tumor with the most colors (five) and the highest score (5 points) on the Seven-Point Checklist algorithm. This tumor had the following colors: black/brown—melanin found in the epidermis, grey—melanin in the superficial dermis, blue—correlated with the deep dermis, and white—fibrosis, collagen located in the dermis [1,10]. Dermoscopic structures: blue-white veil—associated with atypical melanocytes/melanophages located in the dermis [13], irregular dots/globules—melanin located in the epidermis [15], eccentric hyperpigmentation—melanin found at the dermal-epidermal junction (DEJ) [15], regression structures—fibrosis located in the dermis. Based on the dermoscopic findings, the tumor should be located somewhere between the epidermis and the dermis, with a Clark Level of I–II—IB ≤ 1.00–2.00 mm. The histopathological exam showed: Clark Level I, IB = 0.37 mm, see Figure 2—Dermoscopic and histopathological aspects of melanoma.

After we performed the Seven-Point Checklist, according to the algorithm, we had a median of 4.1 points for the confirmed melanomas, with 8 points being the highest score obtained for a superficial spreading melanoma.

### 3.2. Nevi—Dermoscopic Classification

Melanocytic nevi can be classified into the following categories: acquired nevi (dysplastic—junctional/lentiginous, or compound, Spitz, and Reed), congenital nevi (superficial, superficial, and deep, dermal—Miescher (face), and Unna (body) [1]. We classified most of the nevi selected for this study based on their dermoscopic patterns (pattern analysis): reticular, globular, homogenous, and starburst [9].

Two of the selected nevi were acral melanocytic lesions, for which we used the following patterns for analysis: parallel furrow, parallel ridge, lattice-like, and fibrillar; these lesions had a mainly parallel furrow pattern. After the pattern analysis for the rest of the nevi, 48.42% of the tumors (92 nevi) had only one pattern, with the reticular pattern being the most frequent—20%. There were also nevi with two patterns (46.31%, 88 lesions), out of which, the reticular and homogenous nevi were the most encountered—25.78%. Only 4.21% of the nevi (8 lesions) had three patterns (reticular, globular, and homogenous nevi).

- **Atypical nevi—dermoscopic key findings of melanomagenesis?**

Atypical nevi are benign tumors that some studies consider to be precursors of melanoma [16], and it is believed that there could be a linear progression from common to dysplastic nevi that may eventually transform into melanoma under different internal/external factors [16]. It is often difficult to differentiate an atypical nevus from a thin melanoma, due to their clinical and dermoscopic similarities, which is why it is necessary to have an early diagnosis to make the right therapeutic decision (to perform an excision or not, since approximately 30% of this skin cancer is nevus-associated melanomas) [16,17]. To determine if there were any hints of a nevus possibly transforming into a melanoma (hints of melanomagenesis) we assessed the nevi based on the Seven-Point Checklist dermoscopic algorithm (<3 points = nevi, ≥3 points = melanoma—we correlated the results with the lesion possibly being an atypical nevus/candidate for excision). After performing this algorithm, we obtained a median of 0.90 points (compared to the 4.1 median obtained for the confirmed melanomas). We obtained a score of ≥3 points for 21 out of 190 nevi (11.05%), with 4 points being the highest score, whereas most of the nevi had ˂3 points (169 out of 190 lesions—approximately 88.95%). The lesions that had ≥ 3 points were considered atypical nevi and we further analyzed them to see if any other melanoma-specific dermoscopic criteria (white shiny streaks, prominent skin markings, polygons/angulated lines) were present. We obtained the following results: 12 out of 21 lesions (57.14%) presented at least one of the criteria listed above, with polygons/angulated lines being the most predominant criterion (11 out of 21 lesions).

## 4. Discussion

Dermoscopy is an essential tool for the clinician that helps increase the accuracy of melanoma diagnosis, leading to its early detection, and increasing the patient’s chances of survival [3]. Melanoma is an aggressive skin cancer that can arise de novo or on a preexisting nevus [6]. The morphologic heterogeneity of this neoplasm oftentimes makes it difficult to differentiate, especially in its early stages, from certain nevi, as they may have clinical, dermoscopic, or histopathological similarities [16]. For example, in a study by C. Longo et al., there are reports of a rare type of melanoma, nevoid melanoma, that is difficult to differentiate from a nevus, but dermoscopy may provide clues to help identify it [18]. This type of melanoma can be classified as nevus-like, amelanotic, or multi-component type [18]. The most common dermoscopic criteria found in nevoid melanomas are atypical vessels and irregular dots/globules [18].

Melanoma can affect all ages. There are studies that report the occurrence of melanoma at very young ages which are based on both the mutations of certain genes and the expression of carcinogenic risk factors (from intense and unprotected exposure to natural UV radiation, exposure to artificial UV sources, lack of photoprotection, phototype I and II, a high number of nevi, the presence of atypical nevi, and outdoor occupations, to certain oncogenic genotypes of human papillomaviruses) [19].

According to a study by Bernard Ackerman [20], all primary cutaneous melanomas arise in the epidermis, under a sequence of events, with the proliferation of melanocytes that at first extend horizontally along the basal layer and later on extend deeper and deeper into the dermis. Whereas some studies report that nevi are not precursors of melanoma [21], others attest that this is a controversial matter and that a small number of nevi could eventually transform into melanomas through a linear progression influenced by a series of mutagenic factors [22,23]. This linear progression would lead to the occurrence of nevus-associated melanomas (on preexisting nevi) that are reported to develop with a frequency of 20–30%, as the majority of melanomas develop de novo (approximately 70–80%) [4,6,7,8,17,22,23]. However, several studies reported that the histology of nevus-associated melanomas may present an abrupt histologic transition between the benign (nevus) and malignant (melanoma) components [24,25,26]. This may contradict the linear progression theory (the evolution from common/atypical nevi to melanoma). A nevus-associated melanoma is histologically defined by the coexistence of nevus and melanoma components. Two of the main histopathological features of nevi are nesting (melanocytes forming clusters of cells within a tissue) and maturation (progressive change in nest architecture/melanocyte cytology)—characteristics that are usually lost in melanomas [17]. Furthermore, some studies attest that definitive histological features of common and atypical nevi are not observed in the same lesion (nevus-associated melanoma), which suggests that a progression from common to atypical nevi, and later on to melanoma, is probably rare [17]. This theory is strengthened by studies that attest that the risk of a nevus transforming into melanoma over the course of 80 years has been estimated at 0.03% in men and 0.009% in women [7]. A study by Alendar et al. also rejected the theory about tumor progression from common nevi to atypical nevi and then to melanoma, as it was found that melanomas usually develop from a “superficial”/“superficial and deep” congenital nevus, and not from an atypical nevus evolved from a common nevus [27].

As for the results of the study, to analyze the lesions, the Seven-Point Checklist and the pattern analysis algorithms were used. We found the Seven-Point Checklist algorithm to be a very useful tool in distinguishing between nevi and melanomas, as most of the analyzed nevi (169 out of 190 nevi—approximately 88.95%) had under 3 points (benign tumors/nevi), whereas most of the histopathologically confirmed melanomas (8 out of 10 melanomas—80%) had ≥3 points (malignant tumor/melanoma). In a study by Schweizer et al., the Seven-Point Checklist algorithm had an accuracy of 63.9–83.6%, being the second best after the ABCDE rule [28]. Similarly, a study by Argenziano et al. shows that the Seven-Point Checklist has a high sensitivity for melanoma diagnosis [29].

We found the pattern analysis algorithm to be an important tool in classifying nevi, which also draws attention to the atypical morphology of some lesions, especially to those that had two (46.31%) or three patterns (4.21%). In a study by Wolner et al., pattern analysis showed superior diagnostic performance, helping assess the heterogeneity and pattern of the studied lesions [30]. In a study by Seidenari et al., the following dermoscopic criteria were found to be more specific to melanoma in situ, as opposed to atypical nevi: an atypical network that involved more than 75% of the lesion, with more than a type of network, reticular grey-blue areas (78%—MIS vs. 48%—atypical nevi), focal thickening of the network, and a black network [31]. In our study, the most encountered criteria in the atypical nevi population were the polygons/angulated lines (11 out of 21 lesions). The evolution of nevi is a complex process that involves intrinsic and extrinsic factors. Among the different types of nevi, the Spitz nevus is one of the most difficult types to differentiate from melanoma, as it may present itself as a pink-red plaque or nodule (similar to amelanotic melanoma), or it can be pigmented (Reed nevus) [32]. For Spitz nevi/spitzoid lesions, there is yet to be an established consensus regarding their management. As it is oftentimes difficult to differentiate a melanoma from a spitzoid lesion, the following was proposed: for typical Spitz nevi in children under 12 years, the recommendation is for regular clinical follow-up, whereas for lesions that occur after 12 years, they should be excised or digitally monitored until stabilized [33]. All atypical spitzoid lesions, regardless of the age of the patient, should be excised [33].

Regarding the estimation of tumoral thickness, for the studied melanomas, we used dermoscopic colors and specific criteria, and we compared the results to the histopathological examination. The dermoscopic color assessment was not a very accurate criterion for this study as it did not help to establish the exact depth of the tumors; for example, one of the analyzed SSMs had blue colors, which means melanin located in the deep dermis, but the tumor had a histopathologically confirmed Breslow index of 0.5 mm (though it may vary according to the examined situs of the tumor and the experience and knowledge of the pathologist). Our study showed that dermoscopy may not be a very accurate tool for assessing tumoral thickness, but at large, it may help orient the clinician in making the right therapeutic decision. In a study by Martinez-Piva et al., it was reported that dermoscopy is useful in estimating preoperative Breslow thickness in melanoma [13]. In a different study by Sgouros et al., not enough evidence was found regarding dermoscopy estimating the IB of nodular melanoma, but it seems that it may assist in the early recognition of this melanoma subtype [34]. According to a study by Rodriguez-Lomba et al., the accuracy of the combination of dermoscopic colors and structures is yet to be established, but dermoscopy may help in the discrimination between thin and invasive melanomas [35].

Some of the most encountered dermoscopic criteria in the studied melanomas were: polygons (75%—LMM, 40%—SSM), white shiny streaks (100% in LMM, 40%—SSM), blue-white veil (60%—SSM, 75%—LMM), regression structures (80%—SSM, 100%—LMM), rosettes (40%—SSM, 50%—LMM), and irregular hyperpigmented areas (60%—SSM, 100%—LMM). A study by González-Álvarez et al. states that rosettes may be an indicator of incipient melanomas [36], and this may be correct as one of the studied tumors that had rosettes had an IB of 0.37 mm. Concerning the polygons, we found them to be more specific to LMM (75%), similar to a study by Iznardo et al. that highlights that polygons/angulated lines are more frequently seen in lentigo maligna located on the face [37]. All the LMM tumors had white shiny streaks, with them being present in 40% of the studied SSM too, showing that it is a specific dermoscopic structure for melanoma, similar to a study by Verzi et al. that states that white shiny streaks are very specific for melanoma [38]. Blue-white veil and regression structures were some of the most encountered dermoscopic structures that are specific to melanoma. In a study by Martins da Silva, the blue-white veil is mostly found in invasive melanomas [39]. Bassoli et al. stated that identifying regression structures in a tumor is helpful in the diagnosis of early melanoma [40]. In our study, none of the studied tumors that had a blue-white veil or regression structures were invasive melanomas. As for the identification of irregular hyperpigmented areas, in our study, they were found in all the LMM tumors, none of which were invasive melanomas. In a paper published by Lallas et al., irregular hyperpigmented areas seemed to be an indicator of in situ melanoma [41].

One of the article’s limitations is the relatively low number of melanomas which may limit the study’s accuracy. However, we consider this paper to be important as it highlights dermoscopy’s versatility and the many ways in which it can help the clinician, besides being one of the most important tools in skin cancer detection. Another limitation is the research design, which could raise confusion for the audience as it may seem like a mix between a review and an original research article. The intent was to build an original research article, but some aspects had to be discussed (for example, the utility of dermoscopy, Spitz nevi, and various aspects of melanoma) to raise awareness regarding the importance of early melanoma detection and skin cancer prevention. It is very important to diagnose melanoma in its early stages as it is easier to treat, with lower morbidity and mortality rates, as opposed to diagnosing and treating advanced melanomas. In a study by Rotaru et al., most of the studied tumors were diagnosed as advanced melanomas, with an IB of over 2 mm, making the treatment of the patients much more difficult, and associated with low survival rates [42].

Dermoscopy is undoubtedly one of the main tools in early melanoma diagnosis which helps the clinician to better the patient’s prognosis and chances of survival. It is also a great tool for monitoring the patient, especially for individuals with a high risk for melanoma (atypical mole syndrome) [43]. Aside from aiding in diagnosing early skin cancer, dermoscopy also reduces the rates of unnecessary excisions, which leads to cost savings, lower morbidity rates, and less pain and scarring for the patient—facts highlighted in a study by Plüddemann et al. [44]. Moreover, dermoscopy can be used for examining perilesional skin, inflammatory conditions, dermatoses (psoriasis, dermatitis), hair and scalp (trichoscopy), skin infestations and infections (entomodermatoscopy), and nails [1].

## 5. Conclusions

Dermoscopy is a non-invasive and useful tool in the establishment of an early melanoma diagnosis. In addition to classifying melanocytic lesions, it may roughly predict the thickness of tumors based on dermoscopic colors and structures, which may help in choosing the right treatment option. Though tumoral thickness/the Breslow index may be estimated through dermoscopy, the histopathological examination remains superior in this matter. To ease the diagnostic process, the clinician may use certain dermoscopic algorithms, such as the Seven-Point Checklist or pattern analysis. In this study, the Seven-Point Checklist and the pattern analysis algorithms proved to be useful tools for the classification of melanocytic nevi and for differentiating them from melanoma. Early melanoma diagnosis is the key to a better prognosis for the patient as dermoscopy remains an essential tool for dermatologists, with many roles and applications, which helps them read the “messages” left by skin cancers and many other dermatological conditions.

## Figures and Tables

**Table 1:** Melanoma—Dermoscopic findings. “-” = no dermoscopic findings; “+” = positive dermoscopic findings.

Dermoscopic Findings	SSM (Percentage of Dermoscopic Criteria)	NM (Percentage of Dermoscopic Criteria)	LMM (Percentage of Dermoscopic Criteria)
Morphological asymmetry	100%	-	100%
Irregular streaks/pseudopods	40%	-	0%
Atypical network	60%	-	0%
Polygons/angulated lines	40%	-	75%
Blue-white veil	60%	+	75%
Irregular hyperpigmented areas	60%	-	100%
Irregular blotch	0%	-	50%
White shiny streaks	40%	+	100%
Irregular dots/globules	40%	-	100%
Rosettes	40%	-	50%
Regression structures	80%	-	100%
Prominent skin markings	20%	-	0%
Rainbow pattern	20%	-	0%
Crusts/erosions	40%	+	0%
Atypical vascular pattern	0%	-	25%

## Data Availability

The datasets used and/or analyzed during the present study are available from the corresponding author upon reasonable request.
